# FcRn regulates antigen presentation in dendritic cells downstream of DEC205-targeted vaccines

**DOI:** 10.1038/s41541-024-00854-8

**Published:** 2024-04-09

**Authors:** Christophe Macri, Matthew Paxman, Devi Jenika, Xiao Peng Lin, Zahra Elahi, Paul A. Gleeson, Irina Caminschi, Mireille H. Lahoud, Jose A. Villadangos, Justine D. Mintern

**Affiliations:** 1 Department of Biochemistry and Pharmacology, Bio21 Molecular Science and Biotechnology Institute, 30 Flemington Rd, Parkville, The University of Melbourne, Victoria, 3010 Australia; 2 Department of Anatomy and Physiology, Faculty of Medicine, Dentistry and Health Sciences, The University of Melbourne, Parkville, Victoria, 3010 Australia; 3 Department of Microbiology and Immunology, Peter Doherty Institute for Infection and Immunity, The University of Melbourne, Parkville, Victoria, 3010 Australia; 4 Monash Biomedicine Discovery Institute and Department of Biochemistry and Molecular Biology, Monash University, Clayton, Victoria, 3800 Australia

**Keywords:** Antigen presentation, Tumour immunology

## Abstract

Dendritic cell (DC)-targeted vaccination is a new mode of antigen delivery that relies on the use of monoclonal antibodies (mAb) to target antigen to specific DC subsets. The neonatal Fc receptor (FcRn) is a non-classical Fc receptor that binds to immunoglobulin G (IgG) in acidified endosomes and controls its intracellular transport and recycling. FcRn is known to participate in the antigen presentation of immune complexes, however its contribution to DC-targeted vaccination has not previously been examined. Here we have investigated the role of FcRn in antigen presentation using antigen conjugated to IgG mAb which target specific DC receptors, including DEC205 and Clec9A expressed by the conventional DC 1 (cDC1) subset. We show that FcRn is expressed at high levels by cDC1, both at steady-state and following activation and plays a significant role in MHC I cross-presentation and MHC II presentation of antigens that are targeted to cDC1 via mAb specific for DEC205. This effect of FcRn is intrinsic to cDC1 and FcRn impacts the efficacy of anti-DEC205-mediated vaccination against B cell lymphoma. In contrast, FcRn does not impact presentation of antigens targeted to Clec9A and does not regulate presentation of cell-associated antigen. These data highlight a new and unique role of FcRn in controlling the immunogenicity of anti-DEC205-based vaccination, with consequences for exploiting this pathway to improve DC-targeted vaccine outcomes.

## Introduction

Dendritic cells (DCs) are unique in their ability to capture and present antigen by major histocompatibility complex (MHC) molecules to T cells, making them an attractive cellular platform for vaccine development. DC-targeted vaccination is a strategy that relies on delivering the vaccine antigen directly to DCs in situ via conjugation to immunoglobulin G (IgG) monoclonal antibody (mAb) specific to DC surface receptors^1,2^. Several receptors have been explored for DC targeting^1,2^. These include the C-type lectins DC-SIGN and mannose receptor (MR), both expressed by various myeloid cells including monocytes, macrophages and DCs. Antigen delivered to DC-SIGN and MR are efficiently processed for MHC I cross-presentation to CD8^+^ T cell and MHC II presentation to CD4^+^ T cells^3^. Specific DC subsets can also be targeted for vaccination. In this case, antigen is delivered using mAb specific for receptors that possess restricted expression patterns to a particular DC subset of interest. The most widely studied examples are C-type lectin receptors DEC205 and C-type lectin domain family 9 member A (Clec9A), and X-C motif chemokine receptor 1 (XCR1). DEC205, Clec9A and XCR1 are expressed at high levels by conventional DC 1 (cDC1) and few other cell types. cDC1 are a desirable cell type to target for vaccination given their ability to undertake efficient MHC I cross-presentation^4,5^. DEC205 is a receptor for type B oligonucleotides^6^, while Clec9A, also known as DC natural killer lectin group receptor 1 (DNGR1), binds to F-actin exposed by necrotic cells^7–9^. XCR1 is expressed by murine and human cDC1 and recognizes XCR1 ligand XCL1, also known as lymphotactin. DC-targeted vaccination using mAb specific for DEC205, Clec9A and XCR1 induces cytotoxic T cell immunity^10–14^ that contributes to tumor eradication in prophylactic and therapeutic settings^15–17^. In addition, DEC205 and Clec9A-targeted vaccine antigen is efficiently processed for MHC II presentation, resulting in efficient CD4^+^ T cell responses^10,11,13,18^. In contrast to cDC1 receptors, dendritic cell inhibitory receptor 2 (DCIR2), also known as Clec4A4, is a C-type lectin highly expressed by mouse cDC2, a subset specialized in MHC II antigen presentation. DCIR2-targeted vaccination induces poor CD8^+^ T cell responses but generates potent CD4^+^ T cell immunity and robust antibody responses^14,19,20^. DC-targeted vaccines against MR and DEC205 are now used in clinical treatment^21–23^, and efforts to further boost their efficacy is of major interest.

The neonatal Fc receptor (FcRn) is an Fc binding receptor that belongs to the MHC I superfamily, forming a heterodimer encompassing a heavy α chain and a β-2-microglobulin chain^24^. FcRn is predominantly located in endosomal compartments where it interacts with two ligands, IgG and serum albumin via independent binding sites^25^. This receptor-ligand interaction is pH dependent, with relatively strong binding affinity below pH 6.5 but low affinity at neutral pH^26^. FcRn protects IgG and albumin from intracellular catabolism by transporting ligands in transport carriers for release at the cell surface and diverting them away from proteases-containing late endosomes/lysosomes^27^. Another function of FcRn is to deliver IgG from mother to neonates across the intestinal and/or placental epithelia by transcytosis^28,29^. In DCs, FcRn interacts with endocytosed IgG immune complexes and shuttles them to intracellular compartments that enable MHC I and MHC II presentation for priming of CD8^+^ and CD4^+^ T cell responses^30–32^.

Given the role for FcRn in intracellular IgG trafficking and presentation of IgG-associated antigen, we investigated whether FcRn contributes to immune responses downstream of IgG mAb-based DC-targeted vaccines, with a focus on DEC205 and Clec9A-mediated vaccine targeting. Here we show that FcRn plays a critical role in MHC I cross-presentation and MHC II presentation of DEC205-targeted, but not Clec9A-targeted antigen. Our data highlight a critical role for FcRn in settings of DC-targeted vaccination that can be exploited to boost their efficacy.

## Results

### FcRn expression by splenic DCs

**Fig. 1:**
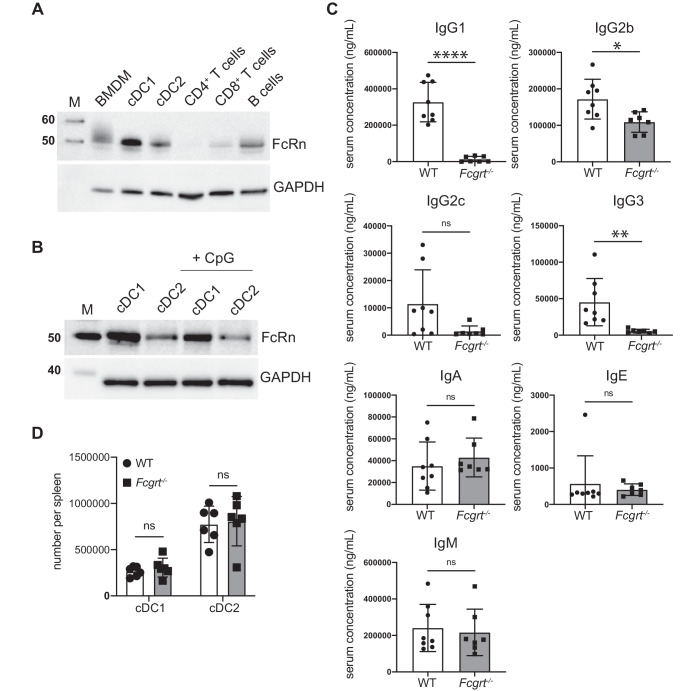
FcRn is expressed by DC subsets. **A** Immune cell populations were purified from C57BL/6 mice and analyzed by immunoblotting for FcRn expression. Representative of two independent experiments. M: Pre-stained protein marker, with the molecular weight indicated in kDa. **B** FcRn expression in spleen cDC1 and cDC2 was analyzed by immunoblotting either in resting conditions or following in vitro activation with CpG for 18 h at 37 °C. M: Pre-stained protein marker, with the molecular weight indicated in kDa. **C** Serum levels of the different IgG subtypes, IgA, IgE and IgM in WT and *Fcgrt*^−/−^ mice. Each symbol represents an individual mouse. ****P < 0.0001, *P < 0.05 by Student’s t test. ns, not significant. **D** The number of spleen cDC1 and cDC2 in WT and *Fcgrt*^−/−^ mice was measured. Each symbol represents an individual mouse. ns not significant.

We first examined FcRn expression in primary mouse cDCs compared to other immune cell types. Total cellular FcRn was detected by western blot for bone marrow-derived macrophages, cDC1, cDC2 and B cells. Low levels of FcRn were detected in CD8^+^ T cells whereas CD4^+^ T cells expressed very little (Fig. 1A). cDC1 and cDC2 express comparable levels of toll-like receptor (TLR)9^33,34^ and both subsets are activated by the TLR9 agonist CpG oligodeoxynucleotides (CpG)^34–36^ (Supplementary Fig. 1). Activation of cDC1 or cDC2 by exposure to CpG did not notably alter FcRn levels for either subset (Fig. 1B). To evaluate FcRn in human DCs, we used the Human DC Atlas^37,38^. *FCGRT* expression was expressed by both human blood-derived cDC1 and cDC2 without stimulation and ex vivo incubation of DCs with bacterial or viral stimuli did not alter *FCGRT* expression for either subset (Supplementary Fig. 2).

We next characterized the phenotype of *Fcgrt*^−/−^ mice. In line with previous reports^27^, *Fcgrt*^−/−^ mice developed hypogammaglobulinemia, as evidenced by the absence of IgG1, IgG2c, IgG3 and a strong reduction in IgG2b levels in the serum. In contrast, IgA, IgE and IgM were detected at normal levels (Fig. 1C). Both cDC1 and cDC2 subsets were present at normal numbers in the absence of FcRn (Fig. 1D).

### FcRn is required for effective tumor immunity in response to DEC205 and Clec9A-targeted vaccination

**Fig. 2:**
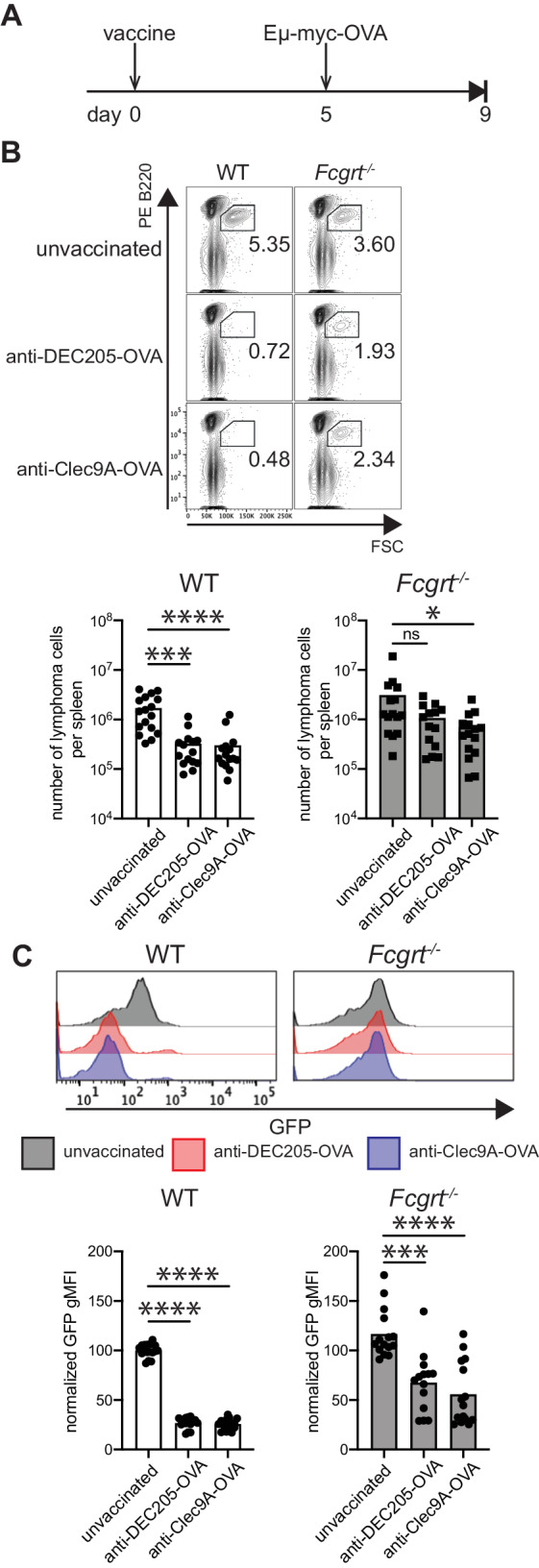
FcRn impacts tumor immunotherapy. **A** WT and *Fcgrt*^−/−^ mice were immunized with anti-DEC205-OVA or anti-Clec9A-OVA mAb together with LPS. After five days, mice were inoculated with Eμ-myc–GFP-OVA tumor cells and four days later, spleens were collected and analyzed by flow cytometry. **B** The contour plots show FSC and B220 expression in whole splenocytes for each group, with the gate indicating the presence of the tumour. The number of tumor cells per spleen is indicated in the histogram. **C** Representative GFP profiles of lymphoma cells for each group. GFP expression level was quantified and shown in the accompanying histogram. Data are pooled from 5 independent experiments with each symbol representing an individual mouse. *P < 0.05. ***P < 0.001, ****P < 0.0001 by Krustal-Wallis with Dunn’s test for multiple comparisons. ns not significant.

The effectiveness of DC receptor-targeted vaccines in eliminating B cell lymphoma was examined using a mouse model. This model consists of Eμ-myc transformed lymphoma cells that express the antigen ovalbumin (OVA)^39^. Prophylactic vaccination is effective in suppressing the growth of Eμ-myc-OVA lymphoma, as shown previously when OVA-coated splenocytes adjuvanted with lipopolysaccharide (LPS) were used as a vaccine^39^. Here, DC vaccines were designed to target whole OVA to DC receptors DEC205 and Clec9A through a genetic fusion of the protein to the Fc region of rat IgG2a specific to the respective receptor. Murine FcRn binds rat IgG2a with high affinity^40^. WT mice were intravenously immunized with either DEC205 or Clec9A-targeted OVA adjuvanted with LPS. Five days later, mice were inoculated with B cell lymphoma and the number of B cell lymphoma cells in the spleen evaluated 4 days later (Fig. 2A). Vaccination with either DEC205 or Clec9A-targeted OVA elicited a significant reduction in tumor cell number, indicating successful anti-tumor therapy for this mode of immunization (Fig. 2B). A role for FcRn was evaluated by undertaking the same analysis in *Fcgrt*^−/−^ mice. In the absence of FcRn, the ability to eradicate tumors was impaired and there was no significant reduction in tumor cell numbers when mice were immunized with DEC205-targeted OVA, while only a mild reduction in tumor load occurred in response to Clec9A-targeted OVA (Fig. 2B). A second measurement of successful tumor immunization was included, whereby evidence of tumor immunoediting in response to vaccination was assessed. In the B cell lymphoma model used here, green fluorescent protein (GFP) is linked by an internal ribosome entry site to the tumor antigen OVA^39^. Therefore, the tumor GFP signal provides a surrogate read out of OVA expression and is a useful marker of tumor antigen expression. In WT mice, DEC205 and Clec9A-targeted OVA immunization results in a significant reduction in tumor GFP. This is likely due to the tumors reducing OVA expression to avoid anti-OVA CD8^+^ T cells elicited by the vaccination regime. In contrast, tumors in *Fcgrt*^−/−^ mice had higher GFP signals, while a significant reduction in GFP signal was still evident compared to tumours in unvaccinated mice. This suggests that in the absence of FcRn there was less pressure on the tumor to edit OVA following immunization (Fig. 2C). Taken together, this data suggests that FcRn plays a pivotal role in promoting the anti-tumor response elicited by DEC205 and/or Clec9A-directed DC-targeted vaccination.

### FcRn regulated MHC I and MHC II antigen presentation in response to DEC205-targeted DC vaccination

**Fig. 3:**
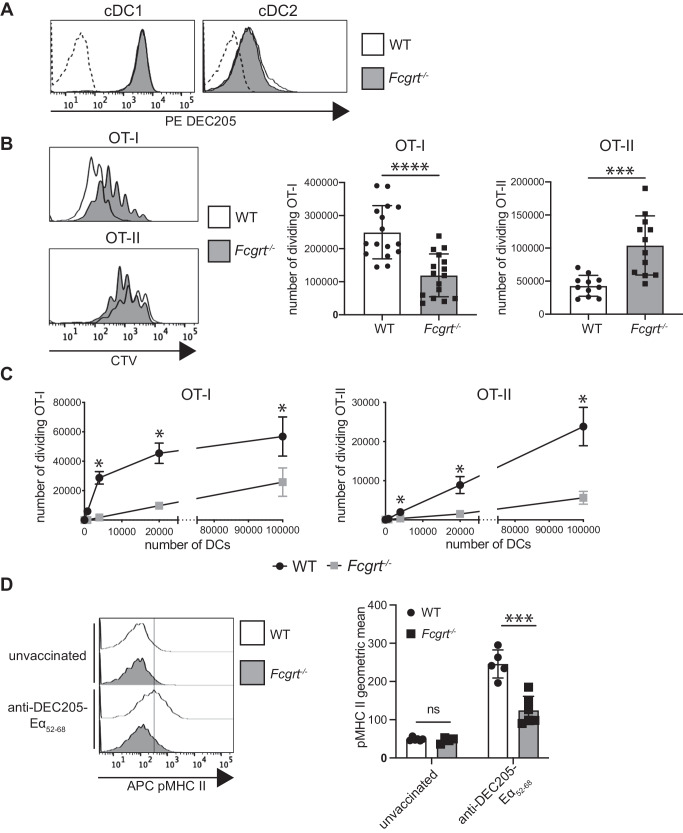
FcRn alters MHC I and MHC II presentation following DEC205-targeted DC vaccination. **A** Flow cytometry analysis of surface DEC205 expression in WT and *Fcgrt*^−/−^ spleen cDCs. Dashed histograms: fluorescence minus one (FMO) background. Data are representative of one out of three analyzed mice. **B** CTV-labelled OT-I and OT-II T cells and CFSE-labelled C57BL/6 mouse splenocytes were adoptively transferred into WT and *Fcgrt*^−/−^ mice. One day later, mice were injected with anti-DEC205-OVA mAb. Spleens were harvested 64 h later and analyzed by flow cytometry. The CTV histograms show OT-I and OT-II proliferation in each group of mice. The number of dividing OT-I and OT-II T cells per spleen was calculated and shown as mean ± SD. Data are pooled from four independent experiments, with 3–4 mice per group. ****P < 0.0001, ***P < 0.001 by unpaired Student’s t-test. **C** WT and *Fcgrt*^−/−^ mice received anti-DEC205-OVA mAb and 20 h later, spleens were harvested and cDC1 purified. Increasing numbers of cDC1 were co-incubated with CTV-labelled OT-I or OT-II at 37 °C for 60 h or 84 h, respectively. The number of dividing OT-I and OT-II cells was quantified by flow cytometry. Data are pooled from two independent experiments, each one done in triplicate. *P < 0.05, by Mann-Whitney test with Holm-Sidak correction for multiple comparisons. **D** WT and *Fcgrt*^−/−^ mice were injected with anti-DEC205-Eα_46-72_ or PBS. Twenty hours later, spleens were harvested. I-Ab-Eα_52-68_ peptide complexes (pMHC II) at the surface of cDC1 were measured by flow cytometry. Histograms are representative of one mouse per group. Data are pooled from two independent experiments with each symbol representing an individual mouse. ***P < 0.001 by unpaired Student’s t-test. ns not significant.

We next examined how FcRn participates in DEC205-targeted DC vaccination. Surface DEC205 was unaltered on splenic cDC1 and cDC2 in the absence of FcRn (Fig. 3A). This finding indicates that similar amounts of DEC205-targeted OVA antigen is delivered to cDCs in both WT and *Fcgrt*^−/−^ mice. Next, the capacity of *Fcgrt*^−/−^ mice to present DEC205-targeted antigen by MHC I and MHC II molecules was examined. To do this, Cell Trace Violet (CTV)-labelled OT-I or OT-II T cells were transferred into WT and *Fcgrt*^−/−^ mice. One day later, mice were immunized with DEC205-targeted OVA intravenously and the number of dividing T cells in the spleen was evaluated by flow cytometry after three days. In the absence of FcRn, DEC205-targeted OVA elicited significantly reduced numbers of divided OT-I, and significantly more divided OT-II cells (Fig. 3B, for gating see Supplementary Fig. 3A). Presentation of OVA-coated splenocytes, used as mAb-independent cell-associated antigen, to OT-I and OT-II was not impacted by FcRn deficiency (Supplementary Fig. 4). To determine if the reduction in MHC I and MHC II antigen presentation was intrinsic to FcRn-deficient cDCs, WT and *Fcgrt*^−/−^ mice were intravenously immunized with DEC205-targeted OVA. Twenty hours later cDC1 were sorted to purity by flow cytometry and equal numbers of WT or *Fcgrt*^−/−^ cDC1s incubated in the presence of CTV labelled OT-I or OT-II T cells. *Fcgrt*^−/−^ cDC1 displayed a significantly reduced capacity to stimulate OT-I and OT-II cells in response to in vivo targeting with DEC205-targeted OVA (Fig. 3C, for gating see Supplementary Fig. 3B). Similarly, cDC2, that express low surface level of DEC205 (Fig. 3A), were significantly impaired in their ability to activate OT-I and OT-II following OVA targeting to DEC205 (Supplementary Fig. 5). This suggests reduced MHC I presentation, and in contrast to in vivo adoptive transfer experiments, reduced MHC II presentation in response to in vivo targeting with DEC205-targeted OVA.

To examine if FcRn impacted presentation of DC-targeted antigen other than OVA, the epitope Eα_46-72_ was targeted to DEC205 and the presence of Ea_52-68_ peptide-loaded I-Ab MHC II molecules at the surface of cDC1 examined using the Yae mAb^41^. WT or *Fcgrt*^−/−^ mice were injected intravenously with DEC205-targeted Eα_46-72_ and cDC isolated from spleen 20 h later. Significantly reduced MHC II presentation of DEC205-targeted Eα_52-68_ was detected at the surface of *Fcgrt*^−/−^ compared to WT cDC1 (Fig. 3D). To further confirm this finding, the *Fcgrt* gene was deleted in the cDC1 cell line MutuDCs^42^ using CRISPR/Cas9 (Supplementary Fig. 6A). Following delivery of the Eα_46-72_ peptide to DEC205 in vitro, *Fcgrt*^−/−^ MutuDCs were significantly impaired in the MHC II presentation of Eα_52-68_ (Supplementary Fig. 6B). Altogether, this data show that FcRn is an important contributor to the response elicited by anti-DEC205-targeted vaccination by promoting MHC I cross-presentation and MHC II presentation of the delivered antigen.

### The role of FcRn in DEC205-targeted DC vaccination is DC-intrinsic

**Fig. 4:**
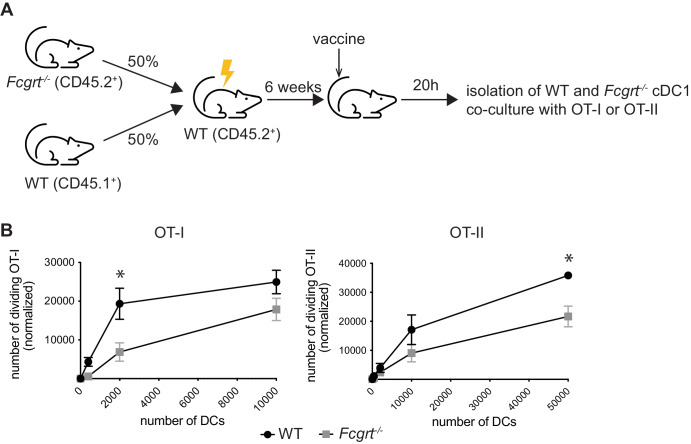
FcRn intrinsically regulate the presentation on antigen targeted to DEC205. **A** WT mice were lethally irradiated and reconstituted with a mix of CD45.1^+^ WT BM and *Fcgrt*^−/−^ (CD45.2^+^) BM. After six weeks, mixed BM chimera mice were injected with anti-DEC205-OVA mAb and 20 h later, spleens were harvested and cDC1 purified. Increasing numbers of cDC1 were co-incubated with CTV-labelled OT-I or OT-II at 37 °C for 60 h or 84 h, respectively. **B** The number of dividing OT-I and OT-II cells was quantified by flow cytometry. Data are pooled from three independent experiments, each one done in triplicate. *P < 0.05 by by Mann-Whitney test with Holm-Sidak correction for multiple comparisons.

One possibility for defects in antigen presentation following DC-targeted vaccination in vivo, is that FcRn is required to promote circulating levels of the DC-targeted IgG vaccine. To address this, we used mixed bone marrow chimeras where WT mice were lethally irradiated and reconstituted with a 1:1 ratio of CD45.1 WT or CD45.2 *Fcgrt*^−/−^ bone marrow. This created a scenario where CD45.1 WT or *Fcgrt*^−/−^ CD45.2 DCs are both in a WT environment with similar levels of circulating DC-targeted IgG vaccine. Six weeks after reconstitution, mice were immunized with anti-DEC205-OVA and 20 h later CD45.1^+^ WT or *Fcgrt*^*−/−*^ cDC1 were isolated from spleens and incubated with CTV labelled OT-I or OT-II. In comparison to WT, *Fcgrt*^*−/−*^ cDC1 displayed significantly reduced MHC I and MHC II presentation of DEC205-targeted OVA (Fig. 4). This rules out that differences in vaccine half-life were responsible for the defects in *Fcgrt*^*−/−*^ cDC presentation of DEC205-targeted antigen and highlights an intrinsic role of FcRn in DCs during anti-DEC205-targeted vaccination.

### FcRn does not regulate MHC I and MHC II antigen presentation in response to Clec9A-targeted DC vaccination

**Fig. 5:**
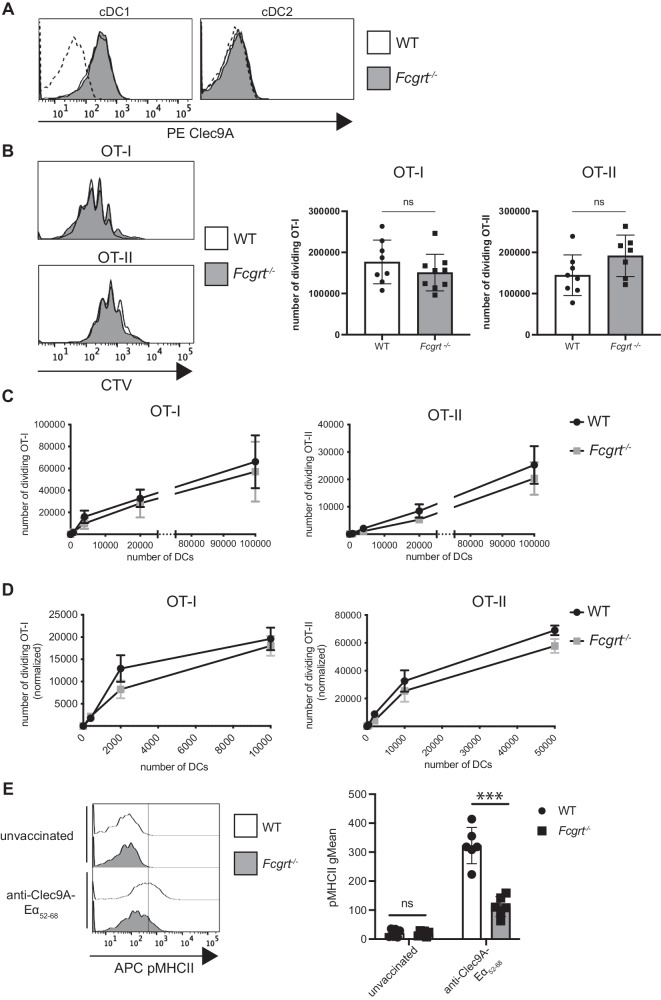
FcRn does not impact MHC I and MHC II presentation following Clec9A-targeted DC vaccination. **A** Flow cytometry analysis of Clec9A expression in WT and *Fcgrt*^−/−^ spleen cDCs. Dashed histograms: FMO background. Data are representative of one out of three mice analyzed. **B** CTV-labelled OT-I and OT-II T cells and CFSE-labelled C57BL/6 mouse splenocytes were adoptively transferred into WT and *Fcgrt*^−/−^ mice. One day later, mice were injected with anti-Clec9A-OVA mAb. Spleens were harvested 64 h later and analyzed by flow cytometry. CTV histograms are representative of OT-I and OT-II proliferation in each group. and the number of dividing OT-I and OT-II T cells per spleen is shown as mean ± SD. Data are pooled from two independent experiments with 3–4 mice per group. ns, not significant by unpaired Student’s t-test. **C** WT and *Fcgrt*^−/−^ mice received anti-Clec9A-OVA mAb and 20 h later, spleens were harvested and cDC1 purified. Increasing numbers of cDC1 were co-incubated with CTV-labelled OT-I or OT-II at 37 °C for 60 h or 84 h, respectively. The number of dividing OT-I and OT-II cells was quantified by flow cytometry. Data are pooled from two independent experiments, each one done in triplicate and analysed by Mann-Whitney test with Holm-Sidak correction. **D** C57BL/6 mice were lethally irradiated and reconstituted with a mix of CD45.1^+^ BM and *Fcgrt*^−/−^ (CD45.2^+^) BM. Mixed BM chimera mice were then treated as in (**C**) and the number of dividing OT-I and OT-II cells was measured. Data are pooled from three independent experiments, each one done in triplicate, and analysed by Mann-Whitney test with Holm-Sidak correction. **E** WT and *Fcgrt*^−/−^ mice were injected with anti-Clec9A-Eα_46-72_ or PBS. Twenty hours later, spleens were harvested. I-Ab-Eα_52-68_ peptide complexes (pMHC II) at the surface of cDC1 were measured by flow cytometry. Histograms are representative of one mouse per group. Data are pooled from two independent experiments with each symbol representing an individual mouse. ***P < 0.001 by Student’s t-test. ns not significant.

Next, we examined how FcRn impacts anti-Clec9A-targeted OVA immunization. Like DEC205, surface Clec9A on cDC1 was not altered by the deficiency in FcRn (Fig. 5A). In contrast to anti-DEC205-OVA mAb, MHC I and MHC II antigen presentation following immunization with anti-Clec9A-OVA mAb was not significantly impacted by the absence of FcRn. This was the case as measured by T cell responses following adoptive OT-I and OT-II T cell transfer into WT and *Fcgrt*^−/−^ mice (Fig. 5B), extrinsic isolation and culture of WT or *Fcgrt*^−/−^ cDC1 from anti-Clec9A-OVA immunized mice (Fig. 5C) and analysis of *Fcgrt*^−/−^ cDC1 compared to WT cDC1 isolated from WT mice reconstituted with 1:1 ratio of WT and *Fcgrt*^−/−^ bone marrow (Fig. 5D). In contrast, MHC II presentation of Clec9A-targeted Eα_46--72_ peptide by cDC1 was significantly reduced in the absence of FcRn (Fig. 5E). Therefore, in contrast to anti-DEC205-targeted vaccination, FcRn does not have a prominent role in regulating the presentation of antigen targeted via Clec9A but can participate under some conditions.

### FcRn is required for CTL immunity to DEC205 but not Clec9A-targeted DC vaccination

**Fig. 6:**
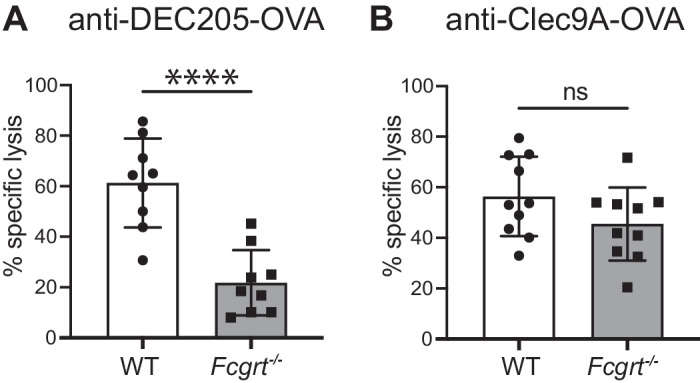
FcRn is required for CTL in response to DEC205, but not Clec9A-targeted DC vaccination. WT and *Fcgrt*^−/−^ mice were vaccinated with anti-DEC205-OVA mAb (**A**) or anti-Clec9A-OVA mAb (**B**), supplemented with LPS as adjuvant. Six days later, mice received an equal number of CTV^hi^ (pulsed with OVA^257-261^ peptide) and CTV^lo^ (unpulsed) target cells. 36–42 h later, spleens were harvested, and the percentage lysis of CTV^hi^ cells was measured by flow cytometry. Data are pooled from two independent experiments with each symbol representing an individual mouse. **** *P* < 0.0001 by Student’s *t*-test. ns not significant.

Elimination of Eμ-myc-OVA B cell lymphoma in response to DC-targeted vaccination likely involves the generation of anti-OVA cytotoxic T lymphocytes (CTL). We therefore examined whether FcRn is required for eliciting CTL in response to anti-DEC205 or Clec9A-OVA mAb. WT or *Fcgrt*^−/−^ mice were intravenously immunized with DC-targeted anti-DEC205 or Clec9A-OVA mAb and six days later the presence of OVA-specific CTL detected by the intravenous injection of OVA_257-264_ peptide-pulsed, or not, target cells. In the absence of FcRn, immunization with anti-DEC205-OVA mAb elicited a significant reduction in CTL lysis compared to WT mice (Fig. 6A, for gating see Supplementary Fig. 7A). In contrast, anti-Clec9A-OVA mAb elicited similar CTL in both WT and *Fcgrt*^−/−^ mice (Fig. 6B). CTL immunity elicited by OVA-coated splenocytes was not impacted by FcRn-deficiency (Supplementary Fig. 7B). Hence, these results demonstrate that priming a robust CTL response following DC-targeted vaccination requires FcRn when delivering antigen to DEC205, but not Clec9A.

## Discussion

DC-targeted vaccination using antigen-conjugated DC-receptor specific IgG mAb allows efficient and precise delivery of vaccine antigen to DC subsets specialized in antigen presentation and T cell priming. The success of this immune therapy relies on the intracellular trafficking pathways inside DCs, that will dictate whether, or not, the vaccine antigen and accompanying targeting IgG can access a compartment that is favourable for antigen presentation. Here we show that mAb-conjugated antigen targeted to DEC205 requires FcRn, a receptor of emerging therapeutic interest^43^, for efficient MHC I cross-presentation and MHC II presentation. In contrast, Clec9A-targeted antigen is presented via a mechanism that is largely independent of FcRn.

Our study highlights a role for FcRn in cDC1. Previously, FcRn was identified as critical for MHC I cross-presentation of immune complex-derived antigen in cDC2, but not cDC1^30,31^. In these systems, the alkaline pH of the cDC1 cross-presentation compartment was not permissive for FcRn binding. In contrast, when antigen is delivered via anti-DEC205 mAb, it accesses late endosomes^44,45^ with a luminal pH that is sufficiently acidic for FcRn binding to the Fc domain of the targeting mAb. In addition to cDC1, other antigen presenting cell populations that express DEC205 may also use FcRn to effectively present DEC205-targeted antigen. For example, B cells which express FcRn, have high surface expression of DEC205^46^ and respond to DEC205 mAb-targeting in vivo by presenting the associated antigen in the context of MHC II^47,48^. Langerhans cells and a subset of dermal DCs also exhibit high surface levels of DEC205. After subcutaneous immunization, these skin DCs capture and transport anti-DEC205 mAb to the draining lymph nodes for presentation of the conjugated antigen to T cells^49,50^. It remains to be determined if FcRn regulates the presentation of DEC205-targeted antigen by these cell types.

FcRn impacts immunization with mAb-conjugated antigen to DEC205, but not Clec9A. Why is this the case? Both DEC205 and Clec9A are expressed at high levels by cDC1, an antigen presenting cell type that we observe expresses high levels of FcRn. Therefore, the different roles for FcRn likely stem from differences in the intracellular trafficking properties of the two receptors. DEC205 and Clec9A exhibit similar plasma membrane internalization patterns in cDC1, with rapid and high surface turnover^51^. Furthermore, we have previously shown that antigen presentation outcomes downstream of antigen targeting to DCs does not correlate with the amount of antigen being endocytosed^51^. Therefore, the difference in requirements for FcRn between antigen targeting to DEC205 versus Clec9A cannot be explained by a different antigen load reaching the endosomal compartment. Inside the cell, however, the receptor pathways diverge. Anti-DEC205 mAbs traffic to late endosomes in immature and mature bone marrow-derived DCs^44,45^ and the highly acidic lumen of these compartments may favour FcRn binding to the Fc-domain of anti-DEC205 IgG. In contrast, Clec9A mAbs transit through early and recycling endosomes^9,52^, compartments without an acidic lumen that may not permit FcRn-IgG binding. Another difference between the two receptors is that mAb binding to Clec9A induces spleen tyrosine kinase (Syk) phosphorylation and endosomal rupture^52^. This has not been reported for DEC205. Therefore, endosomes with antigen-conjugated anti-Clec9A mAb may rupture instead of routing to FcRn-containing endosomes. It is also possible that another binding partner of anti-Clec9A mAb controls its intracellular trafficking in cDC1. Regardless, the impaired presentation of DEC205-targeted antigen when FcRn is absent is likely due to altered trafficking and processing. In cDC2, FcRn directs captured immune complexes to endosomes that are enriched with components of the cross-presentation machinery. These include the translocon Sec61, which mediates the transfer of antigen from the endosomal lumen to the cytosol^53^, and transporter associated with antigen processing 1 (TAP1), which reimports proteasomal generated peptides into the endosome^30^. Interestingly, DEC205-targeted antigen is also cross-presented via a TAP-dependent mechanism^10,54^, suggesting that FcRn may divert anti-DEC205 mAb to similar cross-presenting compartments in cDC1.

Currently, two anti-DEC205 targeting mAbs are in clinical trials. CDX-1401 is a human IgG1 conjugated to the tumor antigen NY-ESO1 that has been evaluated in six phase I and phase II trials^2,21,22^. Another DEC205-specific mAb, DCVax-001 has been conjugated to the human immunodeficiency virus (HIV) antigen Gag p24 and has been tested in a phase I clinical trial^55^. Although these vaccines have shown promising results with robust B cell and T cell immunity, their efficiency may be further enhanced by harnessing FcRn to extend their half-life and activity. Several therapeutic mAb have been modified in their Fc domain to introduce mutations near the key FcRn-binding residues in order to increase the IgG-FcRn binding affinity at low pH. For example, inserting the LS (M428L, N434S) mutation into anti-vascular endothelial growth factor (VEGF) IgG1 Bevacizumab greatly extended mAb half-life by approximately four fold, resulting in increased anti-tumor activity^56^. YTE (M252Y/S254T/T256E) is another mutation introduced into mAb specific for viral proteins. This modification enhanced the half-life of the anti-respiratory syncytial virus IgG1 Motalizumab up to 100 days post-injection^57^. This mAb engineering approach represents a straightforward strategy applicable to DEC205-targeted DC vaccination to improve FcRn affinity and immunogenicity.

In conclusion, our study demonstrates the crucial role of FcRn in the efficient presentation of antigen targeted to DEC205 by DCs. We have shown that FcRn mediates the presentation of DEC205-targeted antigen, while Clec9A-targeted antigen presentation is largely independent of FcRn. The differential trafficking and processing of these receptors likely contributes to the distinct roles of FcRn in antigen presentation of DC-targeted mAb. The clinical trials of anti-DEC205 mAb underscore the potential of utilizing FcRn to enhance the efficacy of DC-targeted vaccines. By engineering the Fc domain of these mAb to increase their affinity for FcRn, the half-life and activity of the vaccines can be extended, leading to improved immunogenicity. These findings provide valuable insights into the development of DC-targeted vaccination strategies and highlight the importance of FcRn in modulating immune responses for effective antigen presentation by DC subsets.

## Methods

### Mice

C57BL/6, *Fcgrt*^*tm1Dcr*^ (*Fcgrt*^−/−^), B6.CH-2^bm-1^ (BM-1), Ly5.1, OT-I x Ly5.1 and OT-II x Ly5.1 mice were used at 6–12 weeks of age. All mice were bred and maintained in specific pathogen–free conditions at the Melbourne Bioresources Platform at Bio21 Molecular Science and Biotechnology Institute. Experimental procedures were approved by the Animal Ethics Committee of the University of Melbourne (protocols 20150 and 21410). Mice were euthanized by carbon dioxide asphyxiation followed by cervical dislocation.

### Isolation of immune cells

For DC purification, spleens were finely chopped and digested in the presence of DNase I (Roche) and collagenase type 3 (Worthington Biochemicals). Intercellular clusters were disrupted by addition of 10 mM Ethylenediaminetetraacetic acid (EDTA). Light density cells were isolated by density gradient separation in 1.077 g/cm^3^ Nycodenz (Nycomed Pharma). Upper fractions were collected, washed and subject to further enrichment by resuspending in a depletion cocktail containing the following rat anti-mouse mAb specific for: CD3 (KT3-1.1; dilution 1:100), CD90 (T24/31.7; dilution 1:100), red blood cells (Ter119; dilution 1:10), B220 (RA3-6B2; dilution 1:100), Ly6G/Ly6C (RB68C5; dilution 1:50) (all Walter and Eliza Hall Antibody Facility). Cells were incubated with antibodies, washed, and incubated with BioMag anti-rat IgG-coupled magnetic beads (Qiagen). The DC-enriched supernatant was recovered by magnetic separation. Where required, DCs were stained with CD11c-FITC (N418; BioLegend Cat. No. 117306; dilution 1:200), CD8α-APC (YTS169.4; Walter and Eliza Hall Antibody Facility; dilution 1:200), CD11b-PE (M1/70; Walter and Eliza Hall Antibody Facility; dilution 1:800) and sorted to purity by flow cytometry on a Becton Dickinson Influx (Murdoch Children’s Research Institute Flow Cytometry and Imaging facility). cDC1 were defined as CD11c^+^ CD8α^+^ CD11b^−^, cDC2: CD11c^+^ CD8α^−^ CD11b^+^. To assess DEC205 and Clec9A expression, cDCs were stained with DEC205-biotin (NLDC-145; Walter and Eliza Hall Antibody Facility; dilution 1:200) followed by streptavidin-APC (BioLegend Cat. No. 405207; dilution 1:500), and Clec9A-APC (7H11; BioLegend Cat. No. 143506; dilution 1:200) and analyzed by flow cytometry. For in vitro activation, cDCs were isolated and incubated overnight in complete RPMI with 0.5 μM type B CpG (Bioneer) at 37 °C or 4 °C. Following activation, cDC subsets were sorted by flow cytometry and analyzed by immunoblotting. Resting and activated cDCs were also stained with CD86-APC (GL1; BioLegend Cat. No. 105012; dilution 1:400), MHC II-Alexa Fluor 700 (M5/114; Walter and Eliza Hall Antibody Facility; dilution 1:400), CD274-APC (10 F.9G2; BioLegend Cat. No. 124312; dilution 1:50) and CD40-FITC (FGK45.5; Walter and Eliza Hall Antibody Facility; dilution 1:100) and analyzed by flow cytometry.

For T cell isolation, single cell suspensions were generated from lymph nodes. Cells were stained with rat anti-mouse mAb specific for: CD11b (M1/70; dilution 1:20), red blood cells (TER119; dilution 1:10), Ly6G (1A8; dilution 1:10), MHC II (M5/114; dilution 1:20), B220 (RA3-6B2; dilution 1:100) and CD4 (GK1.5; dilution 1:10) for CD8^+^ T cells or CD8α (YTS 169.4; dilution 1:25) for CD4^+^ cells (all Walter and Eliza Hall Antibody Facility). Cells were washed and incubated with BioMag anti-rat IgG-coupled magnetic beads (Qiagen). After magnetic depletion, the CD4^+^ or CD8^+^ T cell-enriched supernatant was recovered. Purity was determined by flow cytometry using TCRVα2-APC (B20.1; Walter and Eliza Hall Antibody Facility; dilution 1:1,600) and CD4-FITC (GK1.5; BioLegend Cat. No. 100406; dilution 1:500) or CD8α-FITC (YTS169.4; Walter and Eliza Hall Antibody Facility; dilution 1:1,600). The proportion of CD4^+^ TCRVα2^+^ OT-II cells or CD8α^+^ TCRVα2^+^ OT-I cells was > 90%. For B cell isolation, whole-splenocyte suspensions were subjected to gradient centrifugation (at 2237 *g*) with Ficoll-Paque Plus (GE Healthcare) and subsequent negative depletion using FITC-conjugated mAb against CD4 (GK1.5; dilution 1:300), Ly76 (TER119; dilution 1:300), CD43 (S7; dilution 1:200) (all made by Walter and Eliza Hall Antibody Facility) and magnetic anti-FITC MicroBeads (Miltenyi Biotec). The purity of the B cell preparation was measured by flow cytometry using CD19-FITC (1D3; Walter and Eliza Hall Antibody Facility; dilution 1:800) and B220-PE (RA3-6B2; Beckton Dickinson Cat. No. 553089; dilution 1:200). The proportion of CD19^+^ B220^+^ B cells was > 95%.

### Bone-marrow-derived macrophages

Bone marrow was isolated from the femur and tibia of 6–12-week-old C57BL/6 mice and non-adherent bone marrow cells cultured for 5–7 days in the presence of conditioned media harvested from L-929 cells as a source of macrophage colony-stimulating factor (M-CSF). Purity was assessed by flow cytometry using CD11b-FITC (M1/70; Walter and Eliza Hall Antibody Facility; dilution 1:800) and F4/80-APC (Walter and Eliza Hall Antibody Facility; dilution 1:800). ~95% of live cells express both CD11b and F4/80.

### Immunoblotting

Immune cells were isolated as previously described and were denatured in 1x reducing lithium dodecyl sulfate (LDS) sample buffer (25% (v/v) 4x NuPAGE LDS Sample Buffer (Invitrogen) and 10% (v/v) 10x NuPAGE Reducing Agent (Invitrogen) in distilled water) at 100 °C. Samples were analyzed by sodium dodecyl-sulfate polyacrylamide gel electrophoresis (SDS-PAGE) using 4–12% gradient NuPAGE Bis-Tris polyacrylamide gels (Invitrogen). Proteins were transferred onto Immobilon-P polyvinylidene fluoride (PVDF) membranes (Merck Millipore) and stained with primary antibodies against FcRn (R&D Systems; dilution 1:1,000), glyceraldehyde 3-phosphate dehydrogenase (GAPDH) (6C5; Millipore; dilution 1:2000) followed by horseradish peroxidase (HRP)-conjugated secondary antibodies. Membranes were then incubated with Amersham ECL Plus chemiluminescence reagent (GE Healthcare) for 1 min and protein bands were visualized using the ChemiDoc Imaging System (Bio-Rad). Uncropped and unprocessed blots are shown in Supplementary Fig. 8.

### Serum antibody analysis

For quantification of total serum antibodies, sandwich enzyme-linked immunosorbent assay (ELISA) was performed by coating high protein-binding 96-well plates (Corning) with 20 µg/ml of goat mAb specific for mouse IgA (Cat. No. 1040-01), IgG1 (Cat. No. 1070-01), IgG2b (Cat. No. 1090-01), IgG2a/c (Cat. No. 1080-01), IgG3 (Cat. No. 1100-01), and IgM (Cat. No. 1020-01) and IgE (Cat. No. 1130-01) (all SouthernBiotech). Coated plates were subsequently incubated with diluted serum or standards, followed by detection with HRP-conjugated polyclonal mAb against mouse IgA (Cat. No. 1040-08; dilution 1:1000), IgG1 (Cat. No. 1070-05; dilution 1:500), IgG2b (Cat. No. 1090-05; dilution 1:500), IgG2a/c (Cat. No. 1080-05; dilution 1:500), IgG3 (Cat. No. 1100-05; dilution 1:500), IgM (Cat. No. 1020-05; dilution 1:500) and IgE (Cat. No. 1130-05; dilution 1:500) (all SouthernBiotech) and 1-Step Ultra3,3’,5,5’-Tetramethylbenzidine ELISA Substrate (Thermo Fisher Scientific). Absorbance at 440 nm was measured with a FLUOstar Omega PlateReader, and 4PL analysis was performed with GraphPad Prism software.

### Generating antigen conjugated targeting antibodies

Constructs encoding recombinant DC targeting mAb genetically fused to OVA were generated as previously described in ref. ^13^. For Eα_46-72_ targeting, an antigenic sequence (LESIINFEKLTEEFAKFASFEAQGALANIAVDKANLDVMKEKLPGFGDSIE) containing the epitope Eα_46-72_ (underlined) was genetically fused to the H chain of anti-DEC205 (clone NLDC-145) and anti-Clec9A (clone 10B4) mAb via an alanine linker as described previously^58^. Targeting mAb were expressed using Freestyle 293-F cells and purified by affinity chromatography using protein G sepharose, as previously described^13^.

### Prophylactic vaccination assay and analysis of killing of Eμ-myc-OVA lymphoma

The Eμ-myc lymphoma expressing GFP-OVA was described previously^39^. Mice were immunized i.v. with 1 μg of anti-DEC205-OVA or Clec9A-OVA mAb or 20 × 10^6^ OVA-coated splenocytes, all adjuvanted with 1 μg of LPS. Five days later, mice were challenged with 10^6^ Eμ-myc-OVA lymphoma cells and spleens were harvested after four days. Single-cell splenocyte suspensions were treated with red blood cell (RBC) removal buffer, stained with a B220-PE mAb (RA3-6B2; Beckton Dickinson; Cat. No. 553089; dilution 1:200) and analyzed by flow cytometry.

### In vivo antigen presentation assay

Single-cell suspensions were generated from lymph nodes of OT-I x Ly5.1or OT-II x Ly5.1 mice and the CD8^+^ or CD4^+^ T cells were purified as before. Purified OT-I or OT-II cells were washed with PBS supplemented with 0.1% bovine serum albumin (BSA) and labelled with CTV (Thermo Fisher Scientific). To enable accurate quantification of the number of OT-II or OT-I cells, mice also received transfer of a control population of splenocytes labelled with carboxyfluorescein succinimidyl ester (CFSE, Thermo Fisher Scientific). Single-cell suspensions were treated with RBC removal buffer. Cells were washed with PBS, 0.1% BSA, and labelled with CFSE. Equal numbers of CTV-labelled OT-II or OT-I cells and CFSE-labelled splenocytes were pooled and resuspended in RPMI 1640, 2% (v/v) fetal calf serum (FCS; University of Melbourne Media Preparation Unit) at 1 × 10^7^ cells/ml, respectively, and injected i.v. Twenty-four hours later, mice received 20 × 10^6^ OVA-coated splenocytes, or 0.2 μg anti-DEC205-OVA or anti-Clec9A-OVA mAb via i.v. injection^13^. For OVA-coated splenocytes, single-cell splenocyte suspensions were treated with RBC removal buffer, washed, and incubated with 10 mg/ml OVA protein (Sigma-Aldrich) in FCS-free RPMI 1640 at 37 °C. Sixty-four hours after immunization, spleens were harvested, single-cell suspensions were generated, and RBCs were lysed. Cells were stained with the following mAb: CD8α-APC (53-6.7; BioLegend Cat. No. 100712; dilution 1:800) or CD4-APC (GK1.5; Walter and Eliza Hall Antibody Facility; dilution 1:800), TCRVα2-PE (B20.1; Walter and Eliza Hall Antibody Facility; dilution 1:800) and Ly5.1-PECy7 (A20.1; BioLegend Cat. No. 110730; dilution 1:400). The number of divided OT-II and OT-I was determined as the number of CD4^+^ or CD8α^+^ Ly-5.1^+^ cells that had undergone CTV dilution. The number of cells was normalized to the number of CFSE-labelled splenocytes recovered.

### Ex vivo antigen presentation assay

Mice were immunized i.v. with 1 μg of anti-DEC205-OVA or Clec9A-OVA mAb or anti-DEC205-Eα_46-72_ peptide mAb. After 22–24 h, splenic cDCs were either examined for Eα_52-68_–loaded I-Ab surface expression by flow cytometry using biotinylated Eα_52-68_ mAb (Yae; Thermo Fisher Scientific Cat. No. 13-5741-82; dilution 1:50) and streptavidin-PE (BioLegend Cat. No. 405203; dilution 1:400) or sorted to purity. Sorted cDC1 or cDC2 were cultured in vitro with 5 × 10^4^ CTV-labelled OT-II or OT-I cells in complete medium. Four or five days later, the number of divided OT cells was determined.

### In vitro targeting of MutuDCs and analysis of antigen presentation

MuTu DCs stably expressing Cas9^59^ were cultured in Iscove’s Modified Dulbecco’s Medium (IMDM)-GlutaMax™ (Thermo Fisher Scientific) supplemented with 10% (v/v) (FCS), 100 μM β-mercaptoethanol (Thermo Fisher), 100 μg/mL penicillin and 100 μg/mL streptomycin (University of Melbourne Media Preparation Unit) at 37 °C, 10% CO_2_. To knockout FcRn, the LV04 vector containing the sgRNA was used (Sigma Aldrich). sgRNA sequences were as follows: non-targeting control: 5’ CGCGATAGCGCGAATATATT 3’; FcRn: 5’ CCGTCGGCCCCTCTCCAGG 3’. To produce lentivirus, HEK293T were transfected using polyethylenimine (Polysciences) with sgRNA vector, pMDL (Addgene #12251), pRSV-REV (Addgene #12253), pMD2.G (Addgene #12259). Lentivirus were subsequently used to transduced Cas9^+^ MuTu DCs by spinfection in the presence of polybrene (Sigma-Aldrich). Transduced cells were then selected by culturing them for three days with puromycin (Thermo Fisher Scientific).

### Mixed bone marrow chimera

Recipient mice (C57BL/6) were irradiated twice at 550 cGy (rad), three hours apart before being injected i.v. with 50:50 mixed bone marrow cells from Ly5.1 WT mice and *Fcgrt*^*−/−*^ mice. Recipient mice were intraperitoneally injected with anti-Thy1 mAb (T24, Walter and Eliza Hall Antibody Facility) to eliminate radio-resistant host T cells the day after irradiation. Mice were reconstituted for six weeks before analysis.

### In vivo CTL killing assay

Mice were injected with 1 μg of anti-DEC205-OVA mAb or anti-Clec9A-OVA mAb, or with 2 × 10^7^ OVA-coated splenocytes, together with 1 µg of LPS as adjuvant. Six days later, single-cell suspensions of target cells (splenocytes) were prepared from spleens of WT mice. Half of the suspension (OVA^+^ splenocytes pulsed with OVA_257-264_ labelled with a high concentration of CTV [CTV^hi^]) was pulsed with 1 μg of OVA_257-264_ (Worthington Biochemical) and labelled with 5 mM CTV (Thermo Fisher Scientific). The other half of the suspension (OVA splenocytes labelled with a low concentration of CTV [CTV^lo^]) was labelled with 0.5 mM CTV only. An equal number of OVA^+^ CTV^hi^ and OVA^-^ CTV^lo^ target cells were pooled, and 10 × 10^6^ target cells were injected i.v; 36–42 h later, spleens were harvested and the percentage of OVA-specific lysis was determined as follows: R = (%CTV^lo^ / %CTV^hi^). %OVA-specific lysis = [1 - (*r*_unprimed_ / *r*_primed_)]100.

### Reporting summary

Further information on research design is available in the Nature Research Reporting Summary linked to this article.

### Supplementary information

Supplemental Figures

REPORTING SUMMARY

## Data Availability

Data from this study will be made available upon request to the corresponding author (Justine D. Mintern, jmintern@unimelb.edu.au).
