# Supplementary material for: FcRn regulates antigen presentation in dendritic cells downstream of DEC205-targeted vaccines
Source: NPJ Vaccines. 2024 Apr 9;9:76. doi: 10.1038/s41541-024-00854-8 (PMC11003989; doi:10.1038/s41541-024-00854-8)
Supplement: Supplementary file 1 — Supplemental Figures [file 41541_2024_854_MOESM1_ESM.pdf]

Supplementary Figure 1

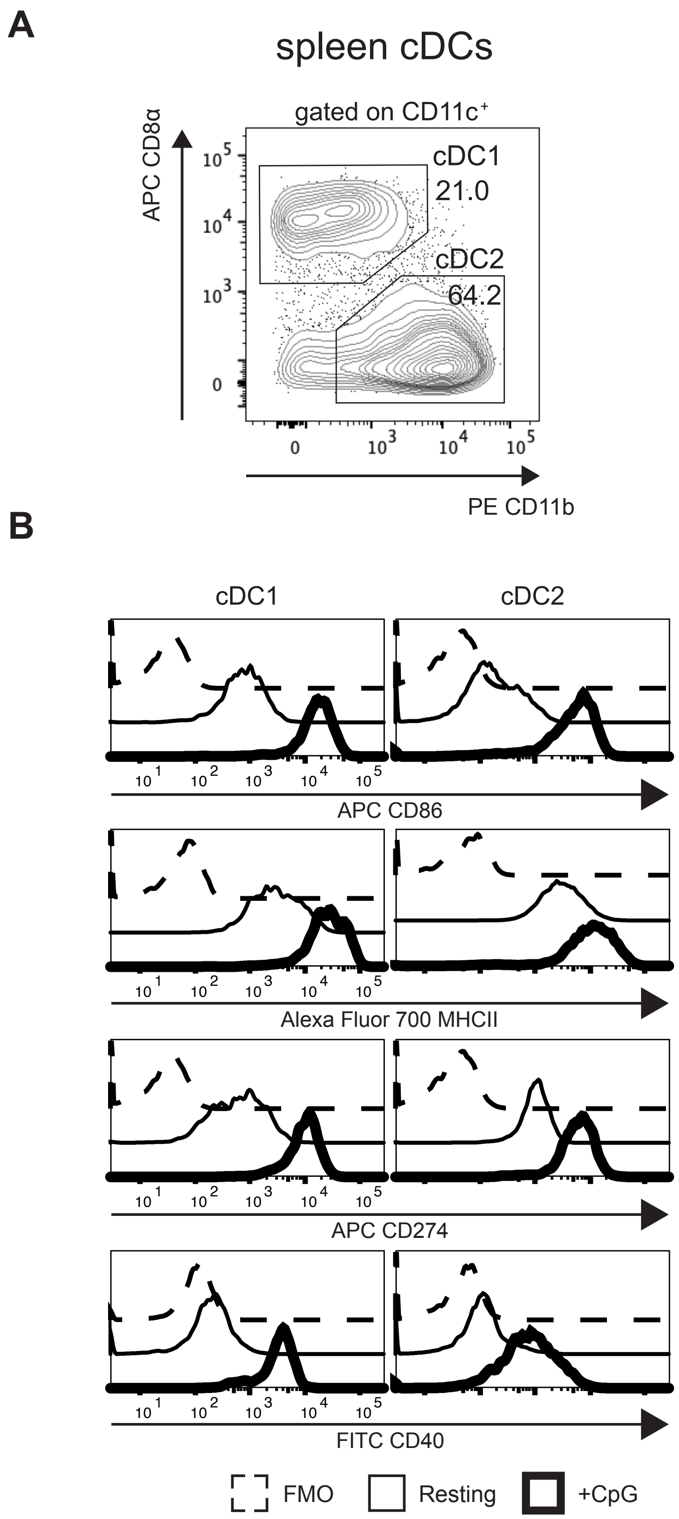

Supplementary Figure 1. Both cDC1 and cDC2 are activated in response to TLR9 stimulation with CpG. A. Purified DCs were gated on forward and side scatter, followed by exclusion of doublets and dead cells (propidium iodide positive). cDC1 are defined as $CD11c^{+}$ $CD8\alpha^{+}$ and cDC2 as $CD11c^{+}$ $CD11b^{+}$. **B.** Flow cytometry analysis of CD86, MHCII, CD274 and CD40 expression in spleen cDCs in resting conditions or following in vitro activation with CpG for 18 hours at 37°C.

Supplementary Figure 2

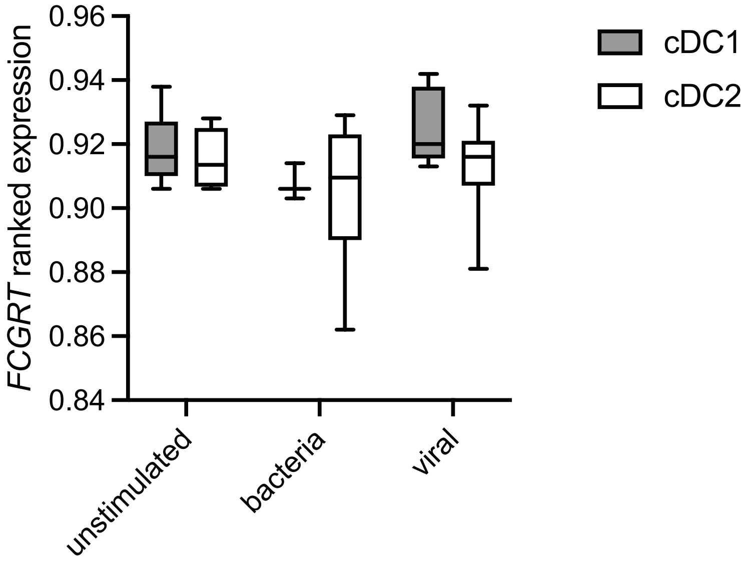

Supplementary Figure 2. FCGRT is expressed in resting and activated human DCs. Ranked expression of *FCGRT* expression in human blood-derived cDC1 and cDC2, incubated *ex vivo* without stimulus or with a bacterial or viral stimulus. Data were obtained from the Human DC Atlas and are represented as box and whiskers plot. The upper and lower limits of the box plot represent the interquartile range and the middle line represents the median. Whiskers represent the maximum and minimum value in each plot.

Supplementary Figure 3

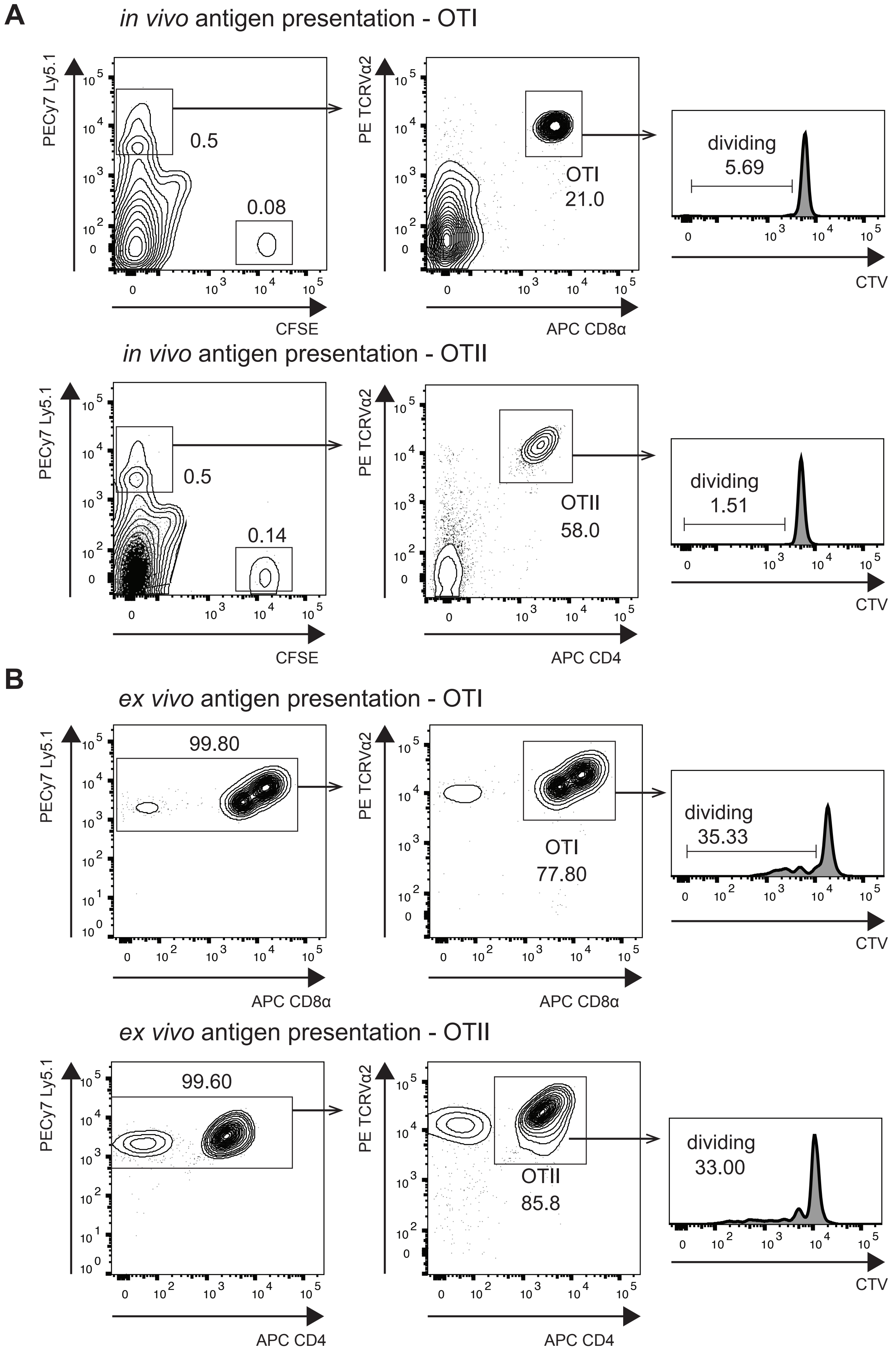

Supplementary Figure 3. Gating strategy for flow cytometry analysis of *in vivo* and *ex vivo* antigen presentation. Cells were gated on forward and side scatter, followed by exclusion of doublets and dead cells (propidium iodide positive). Example of gating is shown for the analysis of OTI and OTII cell proliferation *in vivo* (**A**) and *ex vivo* (**B**).

Supplementary Figure 4

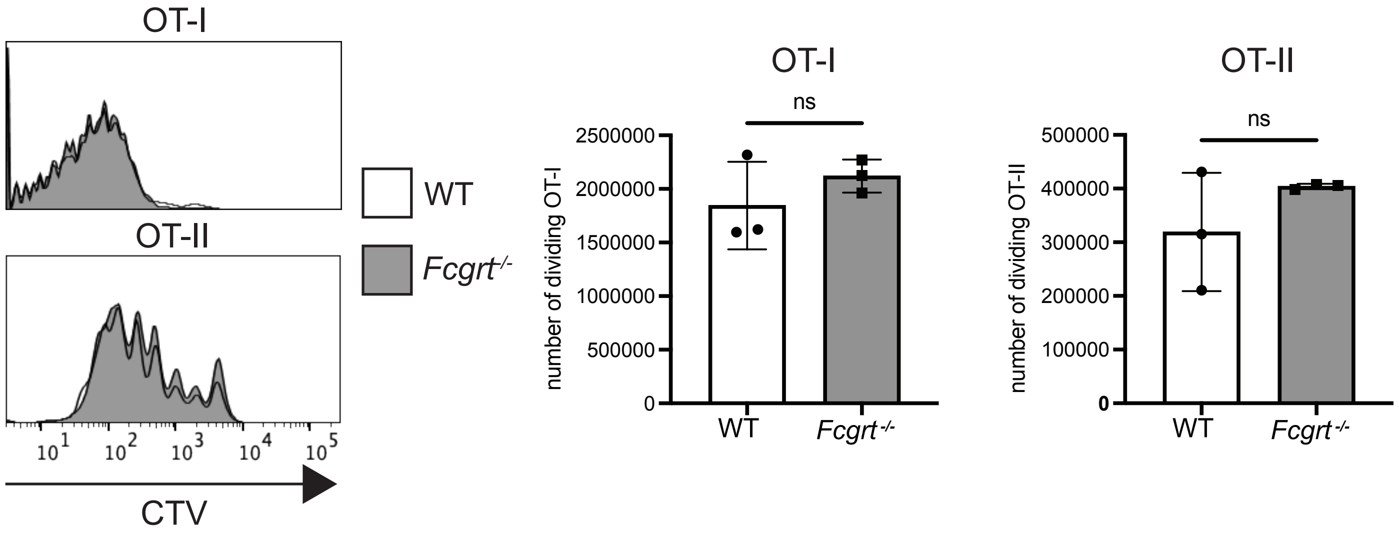

Supplementary Figure 4. FcRn does not impact the MHC I and MHC II presentation following immunization with mAb-independent antigen. CTV-labelled OT-I and OT-II T cells and CFSE-labelled C57BL/6 mouse splenocytes were adoptively transferred into WT and *Fcgrt*$^{-/-}$ mice. One day later, mice were injected with OCS. Spleens were harvested 64 h later and analyzed by flow cytometry. CTV histograms are representative of OT-I and OT-II proliferation in each group. and the number of dividing OT-I and OT-II T cells per spleen is indicated as mean ± SD. Data are pooled from two independent experiments with 3-4 mice per group.

Supplementary Figure 5

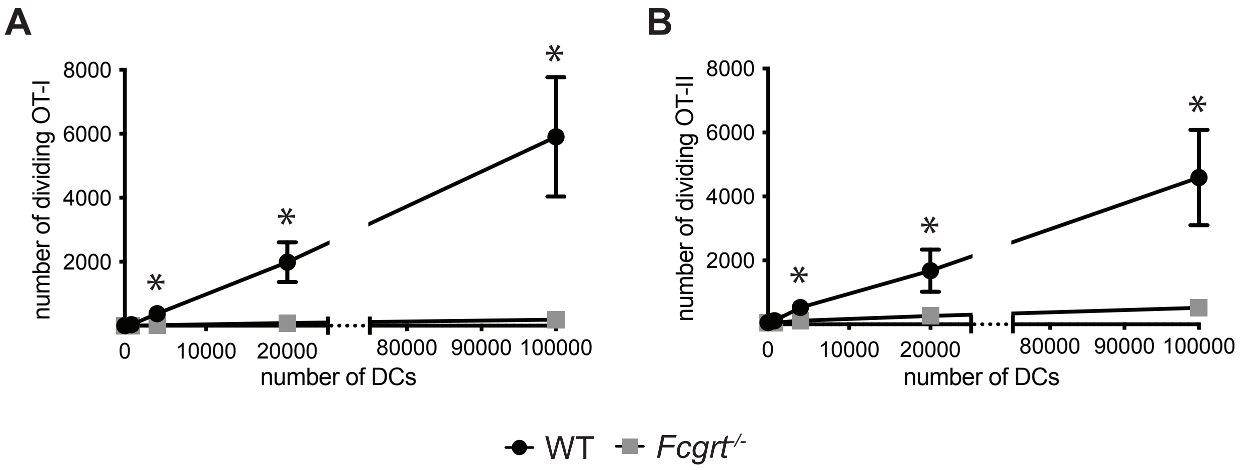

Supplementary Figure 5. FcRn alters MHC I and MHC II presentation in cDC2 following DEC205-targeted DC vaccination. WT and *Fcgrt*$^{-/-}$ mice received anti-DEC205-OVA mAb and 20 hours later, spleens were harvested and cDC1 purified. Increasing number of cDC1 were co-incubated with CTV-labelled OT-I (**A**) or OT-II (**B**) at 37°C for 60 hours or 84 hours, respectively. The number of dividing OT-I and OT-II cells was quantified by flow cytometry. Data are pooled from two independent experiments, each one done in triplicate. * $P < 0.05$, by Mann-Whitney test with Holm-Sidak correction for multiple comparisons.

Supplementary Figure 6

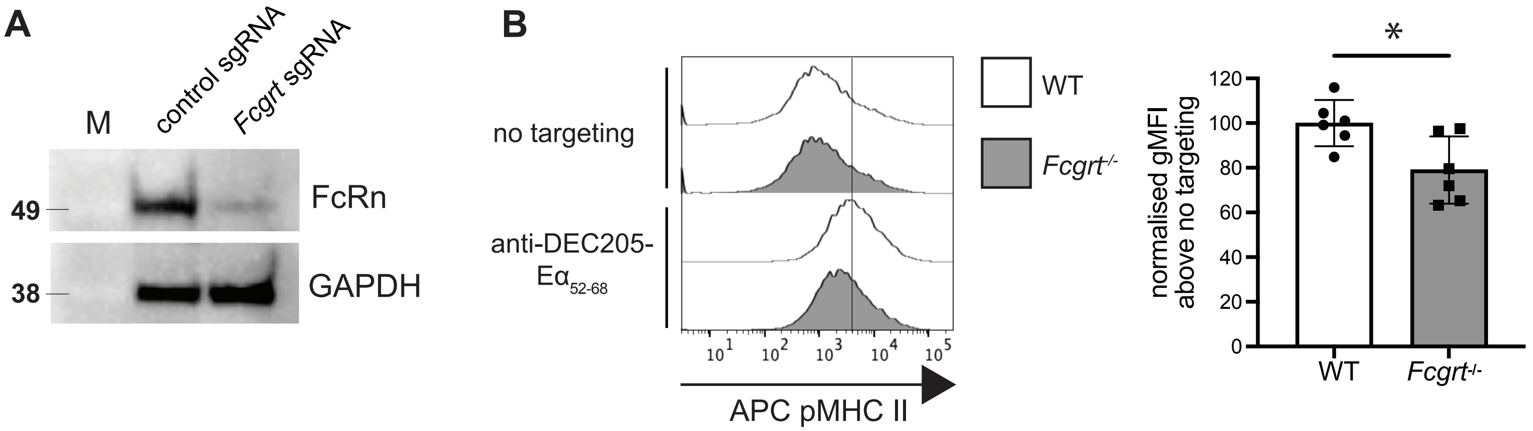

Supplementary Figure 6. FcRn controls the MHC II presentation of antigen targeted to DEC205 on Mutu DCs. **A.** Cas9-expressing Mutu DCs were transduced with lentivirus containing a non-specific gRNA (Control) or a *Fcgrt*-specific gRNA. Cells were analyzed by immunobloting using antibodies specific for FcRn and GAPDH. M: Pre-stained protein marker, with the molecular weight indicated in kDa. **B.** WT and *Fcgrt*$^{-/-}$ Mutu DC were surface labelled with anti-DEC205-E$\alpha_{52\text{-}68}$ or left unlabelled, then washed and incubated at 37°C. Twenty hours later, cells were analyzed by flow-cytometry to detect the presence of I-Ab-E$\alpha_{52\text{-}68}$ peptide complexes (pMHC II) at the plasma membrane using a specific antibody. Data are pooled from two independent experiment, each one done in triplicate. *$P < 0.05$, by Student's t test.

Supplementary Figure 7

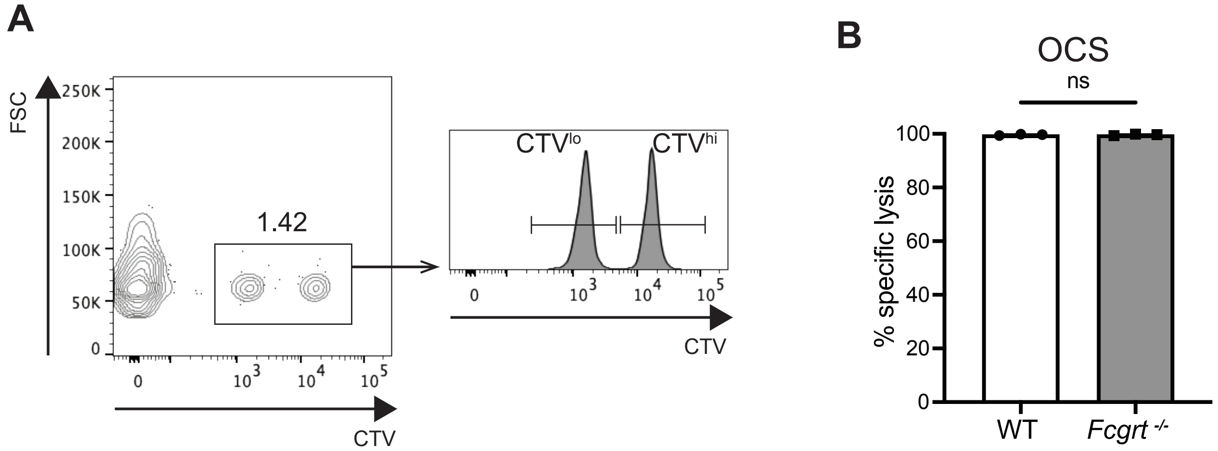

Supplementary Figure 7. Analysis of CTL killing in WT and *Fcgrt$^{-/-}$* mice. A. Gating strategy for flow cytometry analysis of CTL killing, as described in the Material and Methods. **B.** WT and *Fcgrt$^{-/-}$* mice were vaccinated with OCS adjuvanted with LPS. Six days later, mice were injected with equal number of CTV^{hi} (pulsed with $OVA_{257\text{-}261}$ peptide) and CTV^{lo} (unpulsed) target cells. 36-42 h later, the spleens were harvested, and the percentage lysis of CTV^{hi} cells was measured by flow cytometry. Data are pooled from two independent experiments with symbols representing individual mice. ns, not significant by unpaired Student's t test.

Supplementary Figure 8

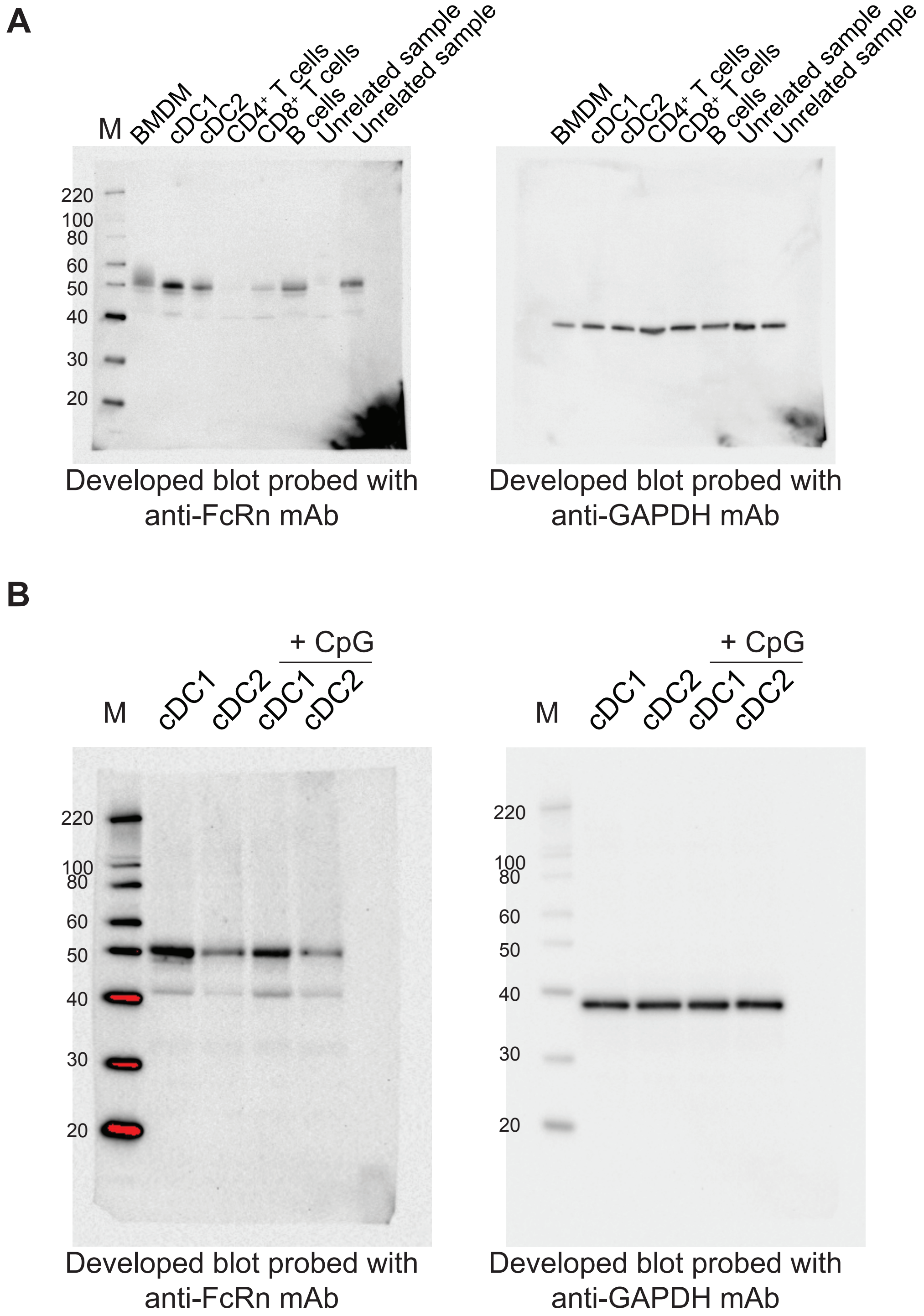

Supplementary Figure 8. Uncropped and unprocessed immunoblots. Immunoblot analysis of C57BL/6 immune cell populations (**A**) and spleen cDC1 and cDC2 in resting conditions or following *in vitro* activation with CpG for 18 hours at 37°C (**B**). The immunoblots were developed using anti-FcRn and anti-GAPDH mAbs. M: Pre-stained protein marker, with the molecular weight indicated in kDa.
