# Supplementary material for: FcRn regulates antigen presentation in dendritic cells downstream of DEC205-targeted vaccines
Source: NPJ Vaccines. 2024 Apr 9;9:76. doi: 10.1038/s41541-024-00854-8 (PMC11003989; doi:10.1038/s41541-024-00854-8)
Supplement: Supplementary file 2 — REPORTING SUMMARY [file 41541_2024_854_MOESM2_ESM.pdf]

nature portfolio

Corresponding author(s): Professor Justine Mintern

Last updated by author(s): Feb 18, 2024

Reporting Summary

Nature Portfolio wishes to improve the reproducibility of the work that we publish. This form provides structure for consistency and transparency in reporting. For further information on Nature Portfolio policies, see our Editorial Policies and the Editorial Policy Checklist.

Statistics

For all statistical analyses, confirm that the following items are present in the figure legend, table legend, main text, or Methods section.

n/a	Confirmed	
☐	☒	The exact sample size (*n*) for each experimental group/condition, given as a discrete number and unit of measurement
☐	☒	A statement on whether measurements were taken from distinct samples or whether the same sample was measured repeatedly
☐	☒	The statistical test(s) used AND whether they are one- or two-sided<br>*Only common tests should be described solely by name; describe more complex techniques in the Methods section.*
☒	☐	A description of all covariates tested
☐	☒	A description of any assumptions or corrections, such as tests of normality and adjustment for multiple comparisons
☐	☒	A full description of the statistical parameters including central tendency (e.g. means) or other basic estimates (e.g. regression coefficient) AND variation (e.g. standard deviation) or associated estimates of uncertainty (e.g. confidence intervals)
☒	☐	For null hypothesis testing, the test statistic (e.g. *F*, *t*, *r*) with confidence intervals, effect sizes, degrees of freedom and *P* value noted<br>*Give P values as exact values whenever suitable.*
☒	☐	For Bayesian analysis, information on the choice of priors and Markov chain Monte Carlo settings
☒	☐	For hierarchical and complex designs, identification of the appropriate level for tests and full reporting of outcomes
☒	☐	Estimates of effect sizes (e.g. Cohen's *d*, Pearson's *r*), indicating how they were calculated

Our web collection on statistics for biologists contains articles on many of the points above.

Software and code

Policy information about availability of computer code

Data collection	Human Dendritic Cell atlas was used to analyze FCGRT gene expression in human dendritic cells.
Data analysis	1. FlowJo was used to analyse flow cytometry data and generate dot plots and histograms (version 10)<br>2. GraphPad Prism was used to generate figures (version 9.5.1)

For manuscripts utilizing custom algorithms or software that are central to the research but not yet described in published literature, software must be made available to editors and reviewers. We strongly encourage code deposition in a community repository (e.g. GitHub). See the Nature Portfolio guidelines for submitting code & software for further information.

Data

Policy information about availability of data

All manuscripts must include a data availability statement. This statement should provide the following information, where applicable:

- Accession codes, unique identifiers, or web links for publicly available datasets
- A description of any restrictions on data availability
- For clinical datasets or third party data, please ensure that the statement adheres to our policy

Data from this study will be made available upon request to the corresponding author (Justine D. Mintern, jmintern@unimelb.edu.au)

Research involving human participants, their data, or biological material

Policy information about studies with human participants or human data. See also policy information about sex, gender (identity/presentation), and sexual orientation and race, ethnicity and racism.

Reporting on sex and gender	n/a
Reporting on race, ethnicity, or other socially relevant groupings	n/a
Population characteristics	n/a
Recruitment	n/a
Ethics oversight	n/a

Note that full information on the approval of the study protocol must also be provided in the manuscript.

Field-specific reporting

Please select the one below that is the best fit for your research. If you are not sure, read the appropriate sections before making your selection.

☒ Life sciences ☐ Behavioural & social sciences ☐ Ecological, evolutionary & environmental sciences

For a reference copy of the document with all sections, see nature.com/documents/nr-reporting-summary-flat.pdf

Life sciences study design

All studies must disclose on these points even when the disclosure is negative.

Sample size	For the in vivo studies, 3-5 mice per group were used.
Data exclusions	No data were excluded.
Replication	The in vivo studies were done in 2-5 independent experiments.<br>Ex vivo antigen presentation studies were done in 2-3 independent experiments, each one done in triplicates.
Randomization	No randomization performed
Blinding	No blinding performed

Reporting for specific materials, systems and methods

We require information from authors about some types of materials, experimental systems and methods used in many studies. Here, indicate whether each material, system or method listed is relevant to your study. If you are not sure if a list item applies to your research, read the appropriate section before selecting a response.

Materials & experimental systems

n/a	Involved in the study	
☐	☒	Antibodies
☐	☒	Eukaryotic cell lines
☒	☐	Palaeontology and archaeology
☐	☒	Animals and other organisms
☒	☐	Clinical data
☒	☐	Dual use research of concern
☒	☐	Plants

Methods

n/a	Involved in the study	
☒	☐	ChIP-seq
☐	☒	Flow cytometry
☒	☐	MRI-based neuroimaging

Antibodies

Antibodies used	Description of commercial antibodies used with clone, company and catalogue number is provided in the Methods.<br>Description of in-house antibodies used with clone name is provided in the Methods.

Validation	All commercial antibodies were validated by their manufacturer<br>All antibodies made by Walter and Eliza Hall Antibody Facility were titrated in house prior to using in experiments.<br>The antigen-conjugated anti-DEC205 and anti-Clec9A antibodies used for vaccination were tested in house on cells that over-express the target and on primary dendritic cells.

Eukaryotic cell lines

Policy information about cell lines and Sex and Gender in Research

Cell line source(s)	MutuDC 1940 cells were a gift from Hans Acha-Orbea, HEK293T cells for lentivirus production were sourced from ATCC
Authentication	Cell lines were authenticated by flow cytometry for expression of expected markers, and morphological analysis.
Mycoplasma contamination	All cell lines were routinely tested for mycoplasma and were negative.
Commonly misidentified lines (See ICLAC register)	None were used

Animals and other research organisms

Policy information about studies involving animals; ARRIVE guidelines recommended for reporting animal research, and Sex and Gender in Research

Laboratory animals	C57BL/6, Fcgrt-/-, B6.CH-2bm-1 (BM-1), Ly5.1, OT-I x Ly5.1 and OT-II x Ly5.1 , age and sex matched (either female or male) were used at 6-12 weeks of age.
Wild animals	Did not involve wild animal
Reporting on sex	Sex was not considered in study design
Field-collected samples	Did not involve field-collected samples
Ethics oversight	Experimental procedures were approved by the Animal Ethics Committee of the University of Melbourne (protocol no. 20150 and 21410).

Note that full information on the approval of the study protocol must also be provided in the manuscript.

Plants

Seed stocks	*Report on the source of all seed stocks or other plant material used. If applicable, state the seed stock centre and catalogue number. If plant specimens were collected from the field, describe the collection location, date and sampling procedures.*
Novel plant genotypes	*Describe the methods by which all novel plant genotypes were produced. This includes those generated by transgenic approaches, gene editing, chemical/radiation-based mutagenesis and hybridization. For transgenic lines, describe the transformation method, the number of independent lines analyzed and the generation upon which experiments were performed. For gene-edited lines, describe the editor used, the endogenous sequence targeted for editing, the targeting guide RNA sequence (if applicable) and how the editor was applied.*
Authentication	*Describe any authentication procedures for each seed stock used or novel genotype generated. Describe any experiments used to assess the effect of a mutation and, where applicable, how potential secondary effects (e.g. second site T-DNA insertions, mosiacism, off-target gene editing) were examined.*

Flow Cytometry

Plots

Confirm that:

☒ The axis labels state the marker and fluorochrome used (e.g. CD4-FITC).

☒ The axis scales are clearly visible. Include numbers along axes only for bottom left plot of group (a 'group' is an analysis of identical markers).

☒ All plots are contour plots with outliers or pseudocolor plots.

☒ A numerical value for number of cells or percentage (with statistics) is provided.

Methodology

Sample preparation	Described in the methods section of the manuscript

Instrument	BD LSR Fortessa
Software	FlowJo version 10
Cell population abundance	For sorted cDCs for western blot analysis and ex vivo antigen presentation assays, the purity was > 95%.<br>The purity of OT-I and OT-II cells used in amtigen presentation assays was > 90%.<br>The purity of B cells used for western blot analysis was > 95%.
Gating strategy	1. SSC-A vs FSC-A to exclude cell debris.<br>2. FSC-H vs FSC-A to include single cells.<br>3. Viability dye (Propidium iodide) vs Fsc-A to exclude dead cells.<br>4. Further gating is described in Supplementary Figure XXX

☒ Tick this box to confirm that a figure exemplifying the gating strategy is provided in the Supplementary Information.
